# Food allergy and anaphylaxis – 2059. Mild symptoms induced by oral food challenge are not always associated with failed challenge results

**DOI:** 10.1186/1939-4551-6-S1-P142

**Published:** 2013-04-23

**Authors:** Taro Miura, Noriyuki Yanagida, Sakura Sato, Yumi Koike, Kiyotake Ogura, Katsuhito Iikura, Takatsugu Komata, Akinori Shukuya, Takanori Imai, Motohiro Ebisawa, Morimitsu Tomikawa

**Affiliations:** 1 Department of Allergy, Sagamihara National Hospital, Kanagawa, Japan; 2 Department of Pediatrics, Natinal Sagamihara Hospital, Kanagawa, Japan; 3 Clinical Research Center for Allergy and Rheumatology, National Sagamihara Hospital, Kanagawa, Japan; 4 Natinal Sagamihara Hospital, Japan; 5 Department of Allergy, Sagamihara National Hospital, Clinical Research Center, Japan; 6 Sagamihara National Hospital, Japan

## Background

Diagnosis of food allergy (FA) is fundamentally based on results of oral food challenge (OFC). In regular practice, we apply open-OFC but not DBPCFC for small children. There seem to be some discrepancy between diagnosis of FA and results of open-OFC. Purpose of this study is to examine correlation between diagnosis of FA and results of OFC.

## Methods

4574 patients (average age 4.0 ± 2.6 years old, male-female ratio 1.89), who had received open-OFC to heated-egg or cow’s milk or wheat from 2005 to 2012, were enrolled to this study. Patients were divided into following 3 categories according to symptoms induced by OFC (primary diagnosis of FA). The “positive” group was patients who showed objective symptoms and “negative” group was patients who had not any symptoms. The third group was defined as “uncertain” who only showed subjective or weak objective symptoms such as slight erythema, mild abdominal pain or isolated cough. Patients with “negative” and “uncertain” group were asked to ingest causative foods or those products at home to confirm whether to induce any symptoms by the intake or not. In several weeks after OFC, we made the final diagnosis based on the information obtained from patients (final diagnosis of FA).

## Results

At primary diagnosis of FA, 29.3% (1343 /4574) patients were categorized as “positive”, 51.7% (2362 /4574) patients “negative” and “uncertain” patients 19.0% (869 /4574). At final diagnosis of FA, 518 of 869 (59.6%) in “uncertain” group was judged as “negative”, whereas 61 of 2362 (2.9%) in “negative” group were decided as “positive” and needed avoid causative foods.

## Conclusions

Although results of OFC are essential for diagnosis of FA, reproducibility of symptoms is important. If patients only show subjective or mild objective symptoms, we need to confirm them regular intake of causative foods.

